# Abdominal wall hematoma as a complication of drainage after laparoscopic cholecystectomy: a case report

**DOI:** 10.3389/fmed.2025.1468200

**Published:** 2025-02-12

**Authors:** Juntao Li, Zixiong Liu, Jia Li, Wei Cheng

**Affiliations:** Department of Hepatobiliary Surgery, Hunan Provincial People’s Hospital, The First Affiliated Hospital of Hunan Normal University, Changsha, China

**Keywords:** abdominal wall hematoma, treatment, laparoscopic surgery, cholecystectomy, complication

## Abstract

**Background:**

Abdominal wall hematoma represents a potential postoperative complication that requires prompt identification and appropriate management. This case report retrospectively analyzes a patient who developed an abdominal wall hematoma associated with a drainage tube and puncture site following laparoscopic cholecystectomy at our hospital. The clinical characteristics, treatment modalities, and relevant literature are reviewed to highlight strategies for the prevention and management of postoperative hematomas, with the aim of providing valuable insights for clinical practice. We managed a patient who had undergone laparoscopic cholecystectomy for gallstones complicated by cholecystitis. On the first postoperative day, a hematoma developed at the site of the abdominal drainage tube insertion. Despite initial attempts at hemostasis through abdominal wall compression, these measures proved ineffective, necessitating the use of a urinary catheter balloon for effective compression hemostasis.

**Case presentation:**

We treated a patient who had undergone laparoscopic cholecystectomy for gallstones complicated by cholecystitis. On the first postoperative day, a hematoma developed at the site of the abdominal drainage tube insertion. Despite initial attempts at hemostasis using abdominal wall compression, these measures were ineffective, necessitating the use of a urinary catheter balloon for effective compression hemostasis.

**Conclusion:**

The urinary catheter balloon tamponade was effectively employed postoperatively to achieve hemostasis for the hematoma at the abdominal wall drainage site. It provides a viable alternative for early intervention in hematoma management.

## 1 Introduction

Laparoscopic surgery is a minimally invasive procedure performed using specialized instruments. Compared to traditional surgery, it presents several advantages, including smaller incisions, greater precision, and reduced intraoperative bleeding. Studies have demonstrated that laparoscopic cholecystectomy does not significantly differ from traditional cholecystectomy in terms of prognosis and complication rates; however, it results in faster postoperative recovery (1).

During abdominal surgery, surgeons typically place a drainage tube to remove accumulated fluid and monitor for potential complications (2). Although the placement of a drainage tube is beneficial for postoperative recovery, it can also give rise to various complications (3), such as drainage site hernias, bleeding, infections, difficulty with tube removal, and bowel obstructions. Among these, bleeding complications generally present as oozing or minimal bleeding. More severe bleeding may be due to incomplete or unreliable hemostasis during the surgery, detachment of vascular ligatures, or pressure ulcers and vascular injuries caused by the drainage tube.

Even though drainage site bleeding is relatively uncommon, if not managed appropriately, it can lead to severe, even life-threatening, consequences. Consequently, timely and appropriate hemostasis is of utmost importance. Bleeding at the drainage site often occurs when excessive negative pressure is applied or when infection or tissue necrosis is present. Abdominal hematomas can be categorized as either intra-abdominal or abdominal wall hematomas, with the latter typically resulting from bleeding in the abdominal wall muscle layer.

If abdominal wall hematomas are not treated promptly and effectively, they can lead to enlargement, infection, and other serious complications, ultimately prolonging hospitalization, increasing healthcare costs, and negatively impacting the patient’s prognosis. Therefore, early identification and intervention in the formation of hematomas are crucial. This study presents a case of an abdominal wall hematoma that developed after laparoscopic cholecystectomy. Despite the patient undergoing traditional abdominal band pressure and suturing treatment following hematoma formation at the drainage site, the hematoma was not effectively controlled. In this particular case, the application of a urinary catheter balloon for hemostasis successfully alleviated the hematoma and prevented further complications.

This report showcases the clinical efficacy and advantages of urinary catheter balloon hemostasis and also delves into strategies for reducing hematoma formation, the causes of abdominal wall hematomas following laparoscopic surgery, and the management approaches for such complications.

## 2 Case presentation

A 61-year-old female patient was admitted to the hospital on August 6, 2020, with a history of recurrent epigastric pain a 1-year history of recurrent epigastric pain, which had relapsed in the past month. The patient described the pain as intermittent, colicky, of moderate intensity, and tolerable. It radiated to the right shoulder and back but was not accompanied by nausea, vomiting, chills, or fever. Despite receiving antispasmodic and anti-infection treatments at a local hospital, the abdominal pain persisted. The patient had no other significant medical history. Upon admission, her vital signs were as follows: heart rate 83 bpm, blood pressure 139/90 mmHg, and normal coagulation function. Abdominal ultrasound revealed gallstones with cholecystitis.

On August 10, 2020, the patient underwent laparoscopic cholecystectomy. During the surgery, the gallbladder appeared congested, edematous, and under high tension, measuring 6 × 4 × 3 cm^3^ with a wall thickness of 3 mm, indicative of subacute inflammation. Intraoperative blood loss was 20 mL. After surgery, the puncture sites were examined, and no significant bleeding was observed following the removal of the puncture sheath. A drainage tube was placed at Winslow’s hole.

On the first postoperative day, 26 hours after surgery, the drainage site dressing showed slight oozing, and the abdominal drainage tube output 20 mL of light red fluid. The patient’s blood pressure dropped to 86/62 mmHg, and hemoglobin decreased to 102 g/L, a drop of 23 g/L from preoperative levels. The hematocrit (HCT) was 32.1%, a decrease of 6% from baseline. Bedside ultrasound showed no significant intra-abdominal fluid collection. The patient was administered 500 mL of hydroxyethyl starch for volume expansion. The drainage dressing was replaced, and the drainage tube was re-sutured with a triangular stitch, but slight oozing persisted, necessitating another dressing change.

**Figure 1:**
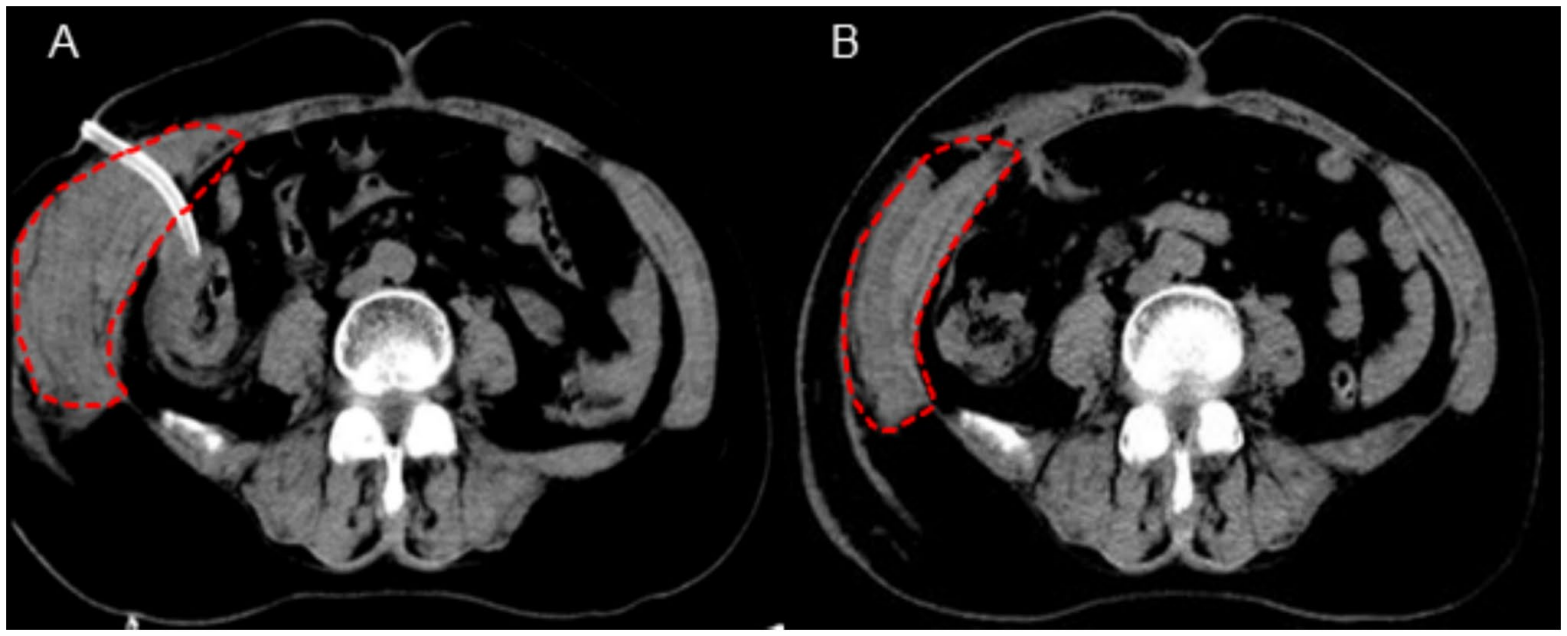
CT imaging findings of the abdominal wall hematoma (**A**: postoperative day 2 evaluation, **B**: postoperative day 5 evaluation).

On the second postoperative day, the patient complained of swelling and mild pain in the right flank and back. After coughing, a small amount of fresh blood drained from the abdominal drainage site. Hemoglobin, rechecked, was 71 g/L, and hematocrit (HCT) was 21.7%. Coagulation function was normal. Physical examination revealed a heart rate of 86 bpm, blood pressure of 102/59 mmHg, and right flank and back swelling, but no abdominal tenderness or signs of peritoneal irritation. An emergency abdominal CT scan revealed thickening of the right abdominal wall soft tissue (Figure 1), with a vertical diameter of 16.3 cm and the thickest part of the muscle layer measuring 5.8 cm, compared to 1.5 cm on the opposite side. The CT density of the abdominal wall soft tissue was 27, while the opposite side had a CT density of 36. A small amount of blood and fluid was noted in the gallbladder region and pelvis. These findings were suggestive of subcutaneous muscle layer hemorrhage and large hematoma formation at the puncture site of the abdominal drainage tube.

**Figure 2:**
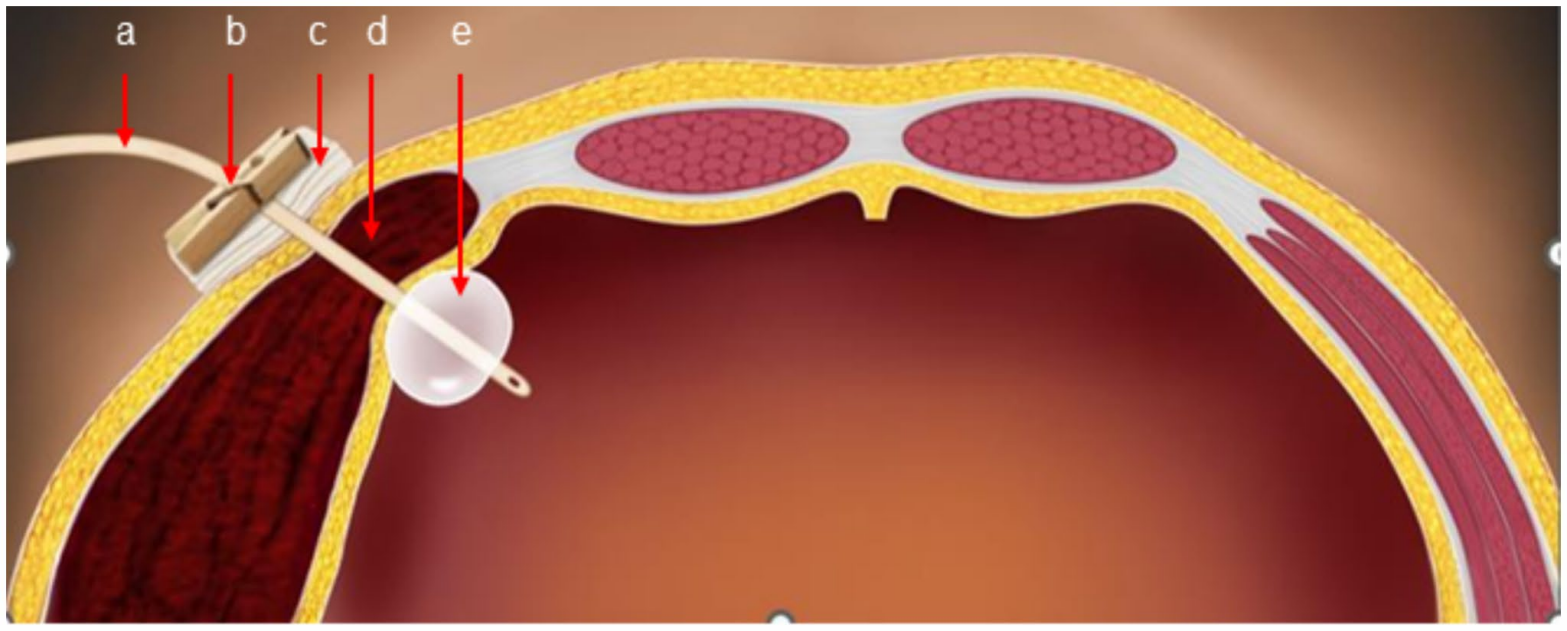
Schematic diagram of catheter balloon compression (a: urinary catheter, b: clamp, c: gauze, d: hematoma, e: catheter balloon).

**Figure 3:**
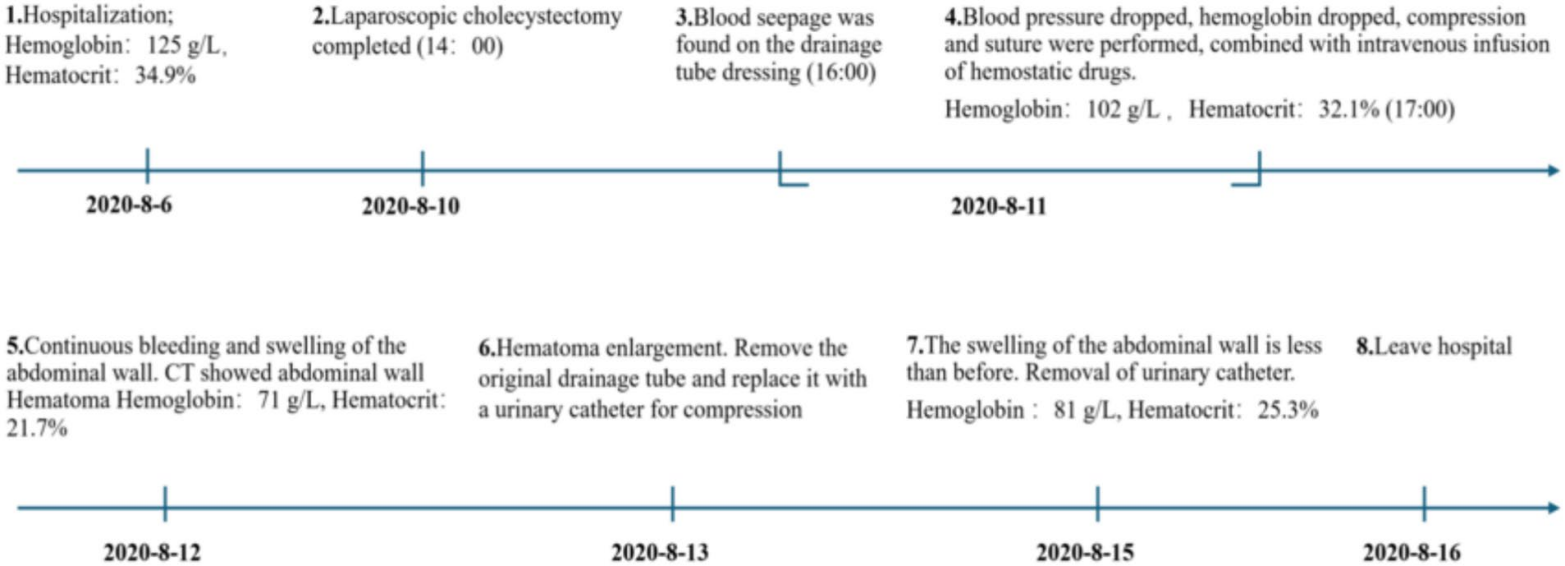
The timeline of the patient’s treatment.

The original abdominal drainage tube was removed, and after sterilization, a urinary catheter was inserted through the fistula into the abdominal cavity. The balloon of the urinary catheter was inflated and retracted to the abdominal wall. After applying slight pressure, the catheter was fixed to the abdominal wall and wrapped with a pressure bandage (Figure 2). Six hours later, repeat hemoglobin and hematocrit tests showed no further decrease, and bedside ultrasound indicated a small amount of pelvic fluid without significant changes. On the fourth postoperative day, the patient developed bruising in the right flank and back, which gradually expanded. On the fifth postoperative day, a repeat CT scan showed a reduction in intra-abdominal blood and fluid, with decreased abdominal wall swelling. The thickest part of the muscle layer measured 3.7 cm, and the vertical diameter was 16 cm. After removal of the urinary catheter and another day of observation, the patient was discharged. The timeline of the patient’s treatment during hospitalization is shown in Figure 3.

## 3 Discussion

In this case, the patient developed early postoperative abdominal wall bleeding and hematoma at the drainage site after laparoscopic cholecystectomy, presumably caused by injury to the abdominal wall artery, potentially related to laparoscopic trocar puncture and the placement of a drainage tube in the abdominal wall. After conservative measures, including pressure dressings and suturing, failed to control the bleeding, a urinary catheter balloon compression technique was chosen as an alternative hemostatic measure. The balloon was inserted through the original drainage site and inflated to apply direct pressure to the bleeding abdominal wall, effectively controlling the hematoma. This method is not commonly reported in routine clinical practice. The inefficacy of traditional hemostatic methods in this case may be due to the deep location of the abdominal wall incision and the large extent of the bleeding. As a result, external pressure and suturing of the superficial incision were insufficient to control the hemorrhage. In clinical practice, for similar cases of abdominal wall bleeding, urinary catheter balloon compression could be considered a targeted intervention. With further research, this method may become a standardized treatment strategy for early postoperative incisional bleeding after laparoscopic surgery.

To minimize the occurrence of bleeding complications, it is essential to carefully select the puncture site when placing the drainage tube and performing abdominal wall puncture. Specifically, areas 4–8 cm from the midline, or within the inner third of the distance between the midline and the anterior superior iliac spine, are high-risk zones for abdominal wall vascular injury. Therefore, during surgery, trocar puncture and drainage tube placement should preferably be done at the midline or laterally, away from these high-risk areas. Recommended puncture points include 1 cm above the midpoint of the line connecting the umbilicus and the upper edge of the pubic symphysis, 1–2 cm to the left or right, the junction of the middle and outer third of the line between the umbilicus and the left anterior superior iliac spine, and the intersection of the umbilical plane and the anterior axillary or midaxillary lines.

Several retrospective studies have investigated the incidence of complications in laparoscopic surgery, showing that the overall incidence of abdominal wall hematomas is less than 2% (Table 1). These studies found that puncture-related complications predominantly occur during the initial puncture of the abdominal wall with a Veress needle (3). One study suggests that using an open technique to establish the first puncture site can reduce the occurrence of laparoscopic-related complications (4). This technique involves creating an abdominal incision under direct visualization to access the abdominal cavity. When creating additional puncture sites with the Veress needle, it is recommended to insert the needle in an avascular area under direct vision, with the puncture direction aimed toward the midline rather than vertically downward (5). If bleeding is observed at the puncture site during surgery, performing full-thickness suturing may mitigate postoperative hemorrhage. Additionally, a hernia needle can be used to perform subcutaneous full-thickness suturing after the procedure. Furthermore, it is essential to carefully select the laparoscopic surgical approach and avoid indiscriminate expansion of surgical indications. Surgeons should enhance their puncture skills and experience through formal surgical training to reduce the risk of complications. If difficulty arises during the procedure or bleeding becomes difficult to control, conversion to open surgery should be considered promptly. Surgeons must be familiar with the characteristics of laparoscopic complications and be prepared to identify and manage them in a timely manner.

**Table 1:** Incidence of abdominal wall hematoma complications in various studies.

References	Year	Study population	Complication
Bhoyrul et al. (10)	2001	629 patients with laparoscopic trocar injuries	Abdominal wall hematoma (*n* = 30, 4.8%)
Mayol et al. (11)	1997	403 patients undergoing laparoscopic surgery	Abdominal wall incision hematoma (*n* = 8, 1.9%)
Triantafyllidis et al. (12)	2009	1,009 patients undergoing laparoscopic cholecystectomy	Abdominal wall hematoma (*n* = 3, 0.30%)
AlKhalifah et al. (13)	2023	510 patients undergoing laparoscopic cholecystectomy	Abdominal wall hematoma (*n* = 1, 0.20%)
Fransen et al. (14)	2012	1,037 patients undergoing single-incision laparoscopic cholecystectomy	Abdominal wall incision hematoma (*n* = 17, 1.6%), periumbilical hematoma (*n* = 5, 0.5%)
Deziel et al. (15)	1993	77,604 patients undergoing laparoscopic cholecystectomy	Abdominal wall hematoma requiring open surgery (*n* = 29, 0.04%)
Z’graggen et al. (16)	1998	10,174 patients undergoing laparoscopic cholecystectomy	Abdominal wall incision hematoma (*n* = 11, 0.11%)

Abdominal wall hematoma is a rare complication following laparoscopic surgery. If persistent bleeding occurs in the abdominal wall, it can pose a life-threatening risk, necessitating timely and effective hemostasis. In the reported cases, clinicians have employed various management approaches depending on the specific clinical situation.

When the patient is hemodynamically stable and no signs of active bleeding are present, conservative treatment may be chosen. This typically includes the use of antibiotics, blood transfusion, and fluid resuscitation, allowing the hematoma to resolve over time (6). If a small amount of active bleeding is observed, manual or instrument-assisted compression can be attempted to achieve hemostasis (7). Conservative treatment and manual compression are among the most commonly used early interventions for hematoma. These methods are favored due to their simplicity and are suitable for minor or superficial abdominal wall bleeding. However, for larger hematomas or deeper bleeding, manual compression is less effective and may lead to continued bleeding if the pressure is insufficient.

In situations where conservative measures fail, or when large hematomas develop or progress to form an abscess, incision and drainage may become necessary (8). Nevertheless, this approach involves invasive procedures and may give rise to complications such as postoperative infection or delayed wound healing.

The utilization of a balloon double-lumen catheter can accurately compress the incision site to control bleeding, serving as an early alternative when conservative treatments and compression techniques prove ineffective. Compared to more invasive interventions like embolization or reoperation, the balloon catheter offers the advantages of being a simple, low-cost procedure with minimal trauma and few complications. The insertion and removal of the catheter are uncomplicated, providing enhanced controllability.

In cases of persistent bleeding and increasing hematoma size despite conventional methods, angiography and embolization are viable options (9). These techniques involve embolizing the target vessels to control bleeding. Additionally, in cases of difficult-to-control hemorrhage, open laparotomy or laparoscopic exploration may be performed to suture the abdominal wall and achieve hemostasis.

Postoperative management of abdominal wall hematoma should be individualized, taking into account factors such as the size of the hematoma, the severity of bleeding, the presence of active bleeding, and the risk of infection.

## 4 Conclusion

This case report emphasizes the effective utilization of a urethral balloon catheter for hemostasis in controlling a postoperative hematoma at the abdominal wall drainage site. When conventional compression and suturing techniques failed to arrest the bleeding, the urethral balloon catheter was adopted as an alternative approach. By exerting consistent and uniform pressure at the original drainage incision site, the balloon successfully achieved hemostasis and mitigated the hematoma.

As an early intervention for hematoma, the urethral balloon catheter effectively controlled bleeding within a short period, thus avoiding more complex interventions or surgical treatments. This approach minimizes tissue damage and reduces the risk of complications. The application of the urethral balloon catheter for hemostasis presents an effective treatment option for the early management of abdominal wall hematoma following laparoscopic surgery, particularly in cases where traditional treatments prove ineffective. With further clinical validation and research, it is anticipated to become a standard recommended treatment for abdominal wall hematomas.

## Data Availability

The raw data supporting the conclusions of this article will be made available by the authors, without undue reservation.
